# Synthesis of Furan-Based Diamine and Its Application in the Preparation of Bio-Based Polyimide

**DOI:** 10.3390/polym15051088

**Published:** 2023-02-22

**Authors:** Yao Zhang, Lei Chen, Yima He, Weiyu Luo, Kaixin Li, Yonggang Min

**Affiliations:** School of Materials and Energy, Guangdong University of Technology, Guangzhou 510006, China

**Keywords:** furan-based diamine, BOC-glycine, bio-based polyimide, synthesis

## Abstract

Furan-based compounds are a new class of compounds characteristic of wide abundance, feasible availability, and environmental friendliness. Presently, polyimide (PI) is the best membrane insulation material in the world, which is widely used in the fields of national defense, liquid crystals, lasers, and so on. At present, most polyimides are synthesized using petroleum-based monomers bearing benzene rings, while furan-based compounds bearing furan rings are rarely used as monomers. The production of petroleum-based monomers is always associated with many environmental issues, and their substitution with furan-based compounds seems a solution to addressing these issues. In this paper, t-butoxycarbonylglycine (BOC-glycine) and 2,5-furandimethanol, bearing furan rings, were employed to synthesize BOC-glycine 2,5-furandimethyl ester, which was further applied for the synthesis of furan-based diamine. This diamine is generally used to synthesize bio-based PI. Their structures and properties were thoroughly characterized. The characterization results showed that BOC-glycine could be effectively obtained using different posttreatment methods. And BOC-glycine 2,5-furandimethyl ester could be effectively obtained by optimizing the accelerating agent of 1,3-dicyclohexylcarbodiimide(DCC) with either 1.25 mol/L or 1.875 mol/L as the optimum value. The PIs originated from furan-based compounds were synthesized and their thermal stability and surface morphology were further characterized. Although the obtained membrane was slightly brittle (mostly due to the less rigidity of furan ring as compared with benzene ring), the excellent thermal stability and smooth surface endow it a potential substitution for petroleum-based polymers. And the current research is also expected to shed some insight into the design and the fabrication of eco-friendly polymers.

## 1. Introduction

Esterification is an essential substitution reaction in organic chemistry and has a wide range of applications in biomedicine, monomer polymerization, etc. It is commonly used in the preparation of pharmaceutical intermediates and as a means of polymerizing monomers into polymers [1]. At the core of the esterification reaction is the dehydroxylation of the carboxyl group through combination with the dehydroxylated hydroxyl groups. Through esterification, several small molecules with bifunctional groups can be polymerized in succession to form polymer compounds, resulting in the preparation of special engineering plastics and rubbers with a variety of physical properties and excellent chemical properties. Esterification was first reported by German chemists Hermann Emil Fischer and Arthur Speier in 1895. Carboxylic acid was employed to react with alcohol under the catalysis of Lewis or Brønsted acid to produce an ester. Based on this reaction mechanism, later extensions and improvements were made, which gradually led to a variety of esterification reactions. Naveed et al. used microbubbles to catalyze the esterification of oleic acid and methanol [2]. B. Ganesh et al. catalyzed the esterification of acetic acid and methanol in a batch reactor [3]. And Zhen et al. catalyzed the esterification of oleic acid and various linear alcohols using magnetic nano-material-immobilized ILs, and good results were achieved [4]. In the esterification reaction, the addition of an accelerant can facilitate the reaction. The commonly used ones are sulfuric acid [5,6], ionic liquid [4,7,8,9,10], solid acid catalyst [11,12,13], ion exchange resin [14,15,16], DCC [17], etc. Among them, DCC can inhibit a large number of side reactions due to its low reaction temperature. Asmaa et al. catalyzed the esterification of propionic acid with different alcohols using DCC/DMAP to obtain propionate at 273 K [18]. Fahmy et al. catalyzed the reaction of lauryl alcohol with methacrylic acid by DCC to obtain methyl lauryl acrylate (LMA), which can only be carried out at room temperature [19]. The advantages of DCC have been fully confirmed in previous work. Therefore, DCC is employed as the accelerator of esterification in this experiment.

In recent years, polyimide (PI) has attracted wide attention as a new type of special engineering polymer material. Because of its great thermal stability and excellent physical properties based on chemical stability [20,21,22], photoresist, membrane, coating, composite material, etc., can be mainly made and is widely applicated in aviation, microelectronic devices, high-temperature resistance, and fireproof materials, electromagnetic devices, liquid crystal displays, etc. [23,24,25,26]. Polyimides can be classified into aliphatic, aromatic, and semi-aromatic groups according to the structure of their repeating units. The aromatic polyimide has a more stable structure due to multitudinous benzene rings and nitrogen heterocycles in the main chain. This structure makes it a superior material with good thermal stability, chemical stability, and excellent physical properties [27]. However, from a green and sustainable point of view, all the mainstream polyimides in the world currently do not have the characteristics of environmental friendliness. Most of the raw materials used in polyimides need to be obtained using industrial synthesis instead of nature, which consumes a lot of fossil energy and tends to cause serious environmental pollution. The reaction of furan compounds yields diamine or dianhydride monomers, which can also be applied to prepare bio-based polyamide. As furan compounds can be directly obtained from the reactions of lignin, sugar, etc., they are widely available in nature, inexpensive and easily accessible, and are excellent biomass-based raw materials [28]. They do not need to undergo the complex industrial preparation that other benzene ring-containing monomers do, thus avoiding environmental pollution and saving energy. As a result, this polyimide is highly environmentally friendly. In this study, the amino group of glycine was protected by BOC and applied to the esterification reaction with 2,5-furandimethanol to prepare BOC-glycine 2,5-furandimethyl ester. After BOC protection was removed, glycine 2,5-furandimethyl ester was obtained. In this process, the influence of different preparation conditions on the structure and yield of the product was investigated. Meanwhile, polyimides were prepared by amidation using 4,4′-diaminodiphenyl ether (ODA) and pyromellitic dianhydride (PMDA) as raw materials [29]. Due to the existence of the furan ring, the polyimide was environmentally friendly [30]. 

## 2. Materials and Methods

### 2.1. Materials

Dioxane(AR), diethyl ether(AR), ethyl acetate (AR), dichloromethane (AR), ethnol (AR), cyclohexane (AR), N,N-dimethylacetamide (DMAc, 99%), 1-methyl-2-pyrrolidinone (NMP, AR), ethanol (AR), K_2_CO_3_ (99%), Na_2_CO_3_ (99%), glycine (98%), 2,5-furandimethanol(98%), 1,3-dicyclohexylcarbodiimide (DCC, 99%), di-tert-butyl dicarbonate ((BOC)_2_O, 99.98%), trifluoroacetic acid (TFA, 98%), 4,4′-diaminodiphenyl ether (ODA, 98%), pyromellitic dianhydride (PMDA, 99%). All the chemicals were used without further purification.

### 2.2. Preparation of BOC-Glycine

According to the literature [31,32], BOC-glycine was synthesized. Under ice bath and mechanical stirring conditions, 0.1 mol glycine and 0.13 mol (BOC)_2_O were added into 35 mL DI water/dioxane (*v*/*v* = 10:25) system. Then, K_2_CO_3_ aqueous solution (0.2 mol K_2_CO_3_ dissolved in 15 mL DI water) was added dropwise using a constant pressure dropping funnel while stirring. Subsequently, it was allowed to continue the reaction until the solution was clear. Then the white solid was obtained after the dioxane and DI water was removed by rotary evaporation under reduced pressure, followed by dissolving in DI water, washed twice with 15 mL ether, then dried with anhydrous sodium sulfate and filtered. Finally, BOC-glycine was obtained using rotary evaporation under reduced pressure. In this step, three different experimental methods were used to treat BOC-glycine after the reaction. In addition to the above methods, we used freeze-drying instead of rotary evaporation, followed by extracting with ethyl acetate after filtration, then dried in vacuum at 60 °C for 12 h to characterize the effects of different treatments.

### 2.3. Preparation of BOC-Glycine 2,5-Furandimethyl Ester

0.05 mol 2,5-furandimethanol, 0.11 mol BOC-glycine and 1.25 mol/L 1,3-dicyclohexylcarbodiimide (DCC) were sequentially added into 40 mL dioxane. After the addition of DCC, the reaction system suddenly boiled, followed by it producing a large amount of white foam, and was then stirred at 90 °C for 24 h. After the reaction, dioxane was removed using rotary evaporation, and then the solid was washed with ethyl acetate. Large quantities of insoluble solids in the system were obtained using filtration, followed by washing with ethanol and being filtered. Filtrate was ultimately dried at 80 °C for 12 h to prepare a yellow solid after rotary evaporation. And then the solid was recrystallized with cyclohexane. In this step, the effect of DCC dosage on the preparation of BOC-glycine 2,5-furandimethyl ester was investigated by changing the dosage of DCC to 0.625 mol/L and 1.875 mol/L, respectively, as experimental variables.

### 2.4. The Removal of BOC Protection

**Scheme 1:**
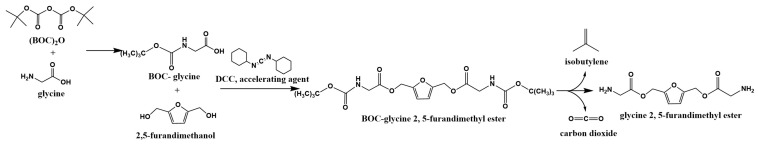
The synthetic route of furan diamine.

According to the reference [33], 18 mL TFA was added into 20 mL dichloroethane to prepare TFA/dichloroethane mixed solution. BOC-glycine 2,5-furandimethyl ester was stirred in TFA/dichloroethane mixed solution at R.T. for 1 h to remove BOC protection, and the viscous black liquid was obtained using rotary evaporation. Then, 5 mL ethyl acetate was added to wash and the pH of the reaction system was adjusted to 8–9 by adding 5% Na_2_CO_3_ aqueous solution. During the addition, bubbles appeared constantly. The system was triphasic after addition, and the solid was obtained after rotary evaporation. The solid was dried at 60 °C for 12 h to prepare glycine 2,5-furandimethyl ester. The synthetic route of furan diamine is shown in Scheme 1.

### 2.5. Preparation of Polyimide Membrane

Glycine 2,5-furandimethyl ester prepared using 1.25 mol/L DCC was also employed in the preparation of polyimide. With the synthesis of 40 g polyimide colloid (20 wt%, the dosage of glycine 2,5-furandimethyl ester was 10 wt%) as an example, 32 g DMAc was added into a 50 mL beaker, then 3.4806 g ODA and 0.3867 g glycine 2,5-furandimethyl ester were added while stirring. Subsequently, 4.1327 g PMDA was added very slowly to prevent violent polymerization. Moreover, the system gradually became viscous with the addition of PMDA. Eventually, the polyimide colloid could be obtained by reacting at room temperature for 12 h at a stirring rate lower than 100 rpm, and the environment should be kept dry during the reaction process. Then, the beaker containing polyimide colloid was put into a vacuum drying dish to remove bubbles. A proper amount of polyimide colloid was coated into a membrane using a spin coater, followed by baking at 90 °C for 30 min and cutting into an appropriate size for imidization reaction. The conditions of imidization were as follows: the membrane was calcined in a muffle furnace at 260 °C for 10 min, then 370 °C for 10 min. Finally, the membrane was taken out after cooling to complete imidization.

### 2.6. Characterization

BOC-glycine, BOC-glycine 2,5-furandimethyl ester, and glycine 2,5-furandimethyl ester were characterized using Fourier transform infrared spectroscopy (FT-IR, Thermofisher Nicolet 6700) in the range of 4000 to 400 cm^−1^, X-ray Diffraction (XRD, D/MAX-Ultima IV) using Cu Kα radiation source (λ = 1.54 Å) at 50 kV and 60 mA, ^1^H-nuclear magnetic resonance (^1^H-NMR, Bruker Avance NEO 400 MHz), ^13^C-nuclear magnetic resonance (^13^C-NMR, Bruker Avance NEO 400 MHz), thermogravimetry (TG, Mettler Toledo TGA/DSC 3+/1600HT), and a liquid chromatography–mass spectrometer (LC–MS, Thermofisher Ultimate 3000 UHPLC-Q Exactive). The polyimide was characterized using gel permeation chromatography (GPC, Thermofisher U3000), thermogravimetry (TG, Mettler Toledo TGA/DSC 3+/1600HT), and a scanning electron microscope (SEM, Zeiss Sigma 300). The scanning range for XRD spectrogram was 10~40° at the rate of 5 °/min. The NMR samples of BOC-glycine were dissolved in D_2_O, the NMR samples of BOC-glycine 2,5-furandimethyl ester and glycine 2,5-furandimethyl ester were dissolved in DMSO-d_6_ and all the samples were tested with the internal standard (Tetramethylsilane, TMS). TG analysis was performed on BOC-glycine, BOC-glycine 2,5-furandimethyl ester, and the samples under nitrogen flow of 50 mL/min, and the stove was heated from room temperature to 500 °C at 10 °C/min. TG analysis of three samples of polyimide membrane was carried out under nitrogen flow of 50 mL/min, and the stove was heated from R.T. to 800 °C at 10 °C/min. The molecular weight of polyimide colloid was analyzed by gel permeation chromatography using NMP solution with 0.05 mol/L LiBr dissolved as the mobile phase at a flow rate of 1.0 mL/min. Molecular weight analysis was performed using a Refracto-Max520 refractive index detector (RI detector) at 3.33 Hz (data collection rate) and the PSS AMA0830101E3 GPC column (300 mm × 8 mm) at 35 °C.

The NMR results of BOC- glycine are shown below: BOC- glycine prepared by freeze-drying (400 Hz, D_2_O): ^1^H-NMR: 3.42 (d, 2H), 1.30 (s,9H). ^13^C-NMR: 31.72 (s), 45.50 (s), 81.24 (s), 170.73 (s). BOC- protected glycine prepared by rotary evaporation (400 Hz, D_2_O): ^1^H-NMR: 3.46 (d, 2H), 1.29 (s, 9H). ^13^C-NMR: 31.75 (s), 45.45 (s), 81.31 (s), 170.75 (s). BOC- glycine prepared by extracting with ethyl acetate after filtration and drying in vacuum (400 Hz, D_2_O): ^1^H-NMR: 3.40 (d, 2H), 1.30 (s,9H). ^13^C-NMR: 31.72 (s), 45.51 (s), 81.26 (s), 170.85 (s).

The NMR results of BOC-glycine 2,5-furandimethyl ester are shown below: BOC-glycine 2,5-furandimethyl ester prepared by using 1.875 mol/L DCC (400 Hz, (CD_3_)_2_SO, yield:80%): ^1^H-NMR: 6.32 (t, 1H), 5.57 (d, 2H), 5.31 (s, 1H), 3.55 (d, 2H), 1.37 (s, 9H). ^13^C-NMR: 33.82 (s), 47.96 (s), 62.02 (s), 82.77 (s), 116.29 (s), 157.14 (s), 158.40 (s), 161.90 (s). After BOC removal (400 Hz, (CD_3_)_2_SO, yield:76%): ^1^H-NMR: 6.17 (t, 2H), 5.58 (d, 2H), 5.32 (s, 1H), 2.95 (d, 2H), 1.98 (m, 2H), 1.50 (dd, 4H). ^13^C-NMR: 47.97 (s), 66.84 (s), 119.28 (s), 154.19 (s), 158.36 (s), 160.31 (s). BOC-glycine 2,5-furandimethyl ester prepared by using 1.25 mol/L DCC (400 Hz, (CD_3_)_2_SO, yield:81%): ^1^H-NMR: 6.31 (t, 1H), 5.57 (d, 2H), 5.32 (s, 1H), 3.57 (d, 2H), 1.38 (s, 9H). ^13^C-NMR: 33.81 (s), 47.97 (s), 60.84 (s), 82.54 (s), 116.53 (s), 157.14(s), 158.40 (s), 161.90 (s). After BOC removal (400 Hz, (CD_3_)_2_SO, yield:78%): 6.17 (t, 2H), 5.57 (d, 2H), 5.33 (s, 1H), 2.67 (d, 2H), 1.72 (m, 2H), 1.25 (dd, 4H). ^13^C-NMR: 47.82 (s), 66.84 (s), 116.53 (s), 155.19 (s), 158.40 (s), 160.75 (s). BOC-glycine 2,5-furandimethyl ester prepared by using 0.625 mol/L DCC (400 Hz, (CD_3_)_2_SO): ^1^H-NMR: 6.17 (s, 1H), 5.31 (s,1H), 1.46 (s, 9H). ^13^C-NMR: 33.82 (s), 47.97 (s), 153.02 (s), 159.08 (s). After BOC removal (400 Hz, (CD_3_)_2_SO): ^1^H-NMR: 3.54 (d, 1H), 2.16 (s, 1H), 1.94 (p, 4H). ^13^C-NMR: 47.57 (s), 153.92 (s), 157.07 (s).

## 3. Results and Discussion

### 3.1. Characterization of BOC-Glycine and Ester

**Figure 1:**
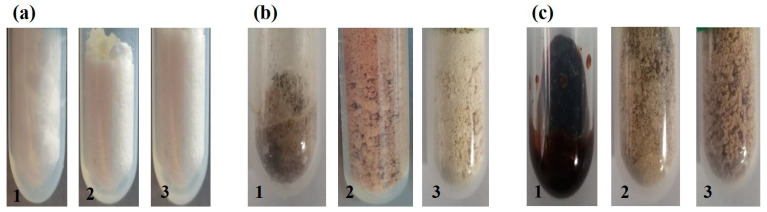
(**a**) Three kinds of BOC-glycine. Three kinds of BOC-glycine 2,5-furandimethyl ester using different dosages of DCC (**b**) before and (**c**) after BOC removal: (**1**) 0.625 mol/L. (**2**) 1.25 mol/L. (**3**) 1.875 mol/L.

The appearance of all BOC-glycine, glycine 2,5-furandimethyl ester before and after BOC removal is shown in Figure 1. Figure 1a shows that the appearance of BOC-glycine prepared using the three methods was white and powdery, which was essentially the same as the BOC-glycine currently available on the market. Figure 1b showed the BOC-glycine 2,5-furandimethyl ester prepared using three different DCC dosages. It could be seen directly from the appearance that the color of the obtained solid would gradually lightened with the increase in DCC dosage, which maybe because the higher amount of DCC dosage that was used in the reaction, the more 1,3-dicyclohexylurea (DCU) that was produced from DCC dehydration in the esterification reaction. Additionally, DCU is a white crystalline powder that may lighten the color of the obtained solid. The appearance of glycine 2,5-furandimethyl ester prepared by using 1.25 mol/L DCC and 1.875 mol/L DCC showed similar dark yellow solids, as shown in Figure 1c. However, the sample prepared by using 0.625 mol/L DCC only yielded a black viscous liquid rather than a solid after rotary distillation. Extending the time of rotary distillation was not effective and only resulted in no more solids or liquids remaining in the reaction system.

**Figure 2:**
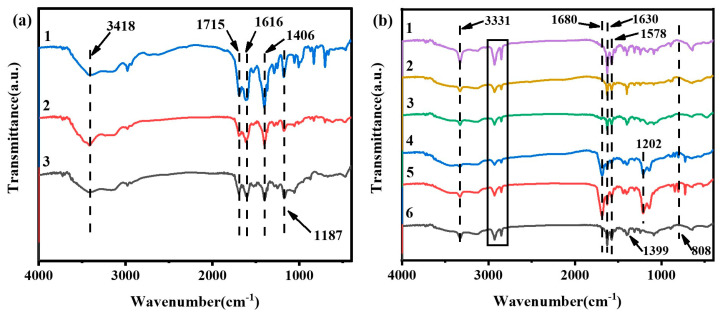
FTIR spectra of all prepared BOC-glycine and esters. (**a**) **1.** Freeze-drying. **2.** Vacuum distillation. **3.** Extracted with ethyl acetate after filtration and dried in vacuum. (**b**) **1** and **4.** Ester used 1.875 mol/L DCC before and after BOC removal. **2** and **5.** Ester used 1.25 mol/L DCC before and after BOC removal. **3** and **6.** Ester used 0.625 mol/L DCC before and after BOC removal.

**Figure 3:**
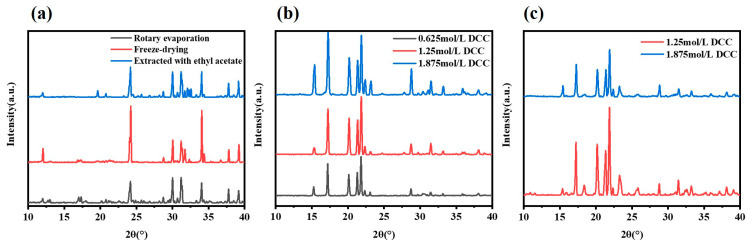
XRD spectra of all prepared BOC-glycine and esters. (**a**) BOC-glycine. (**b**,**c**) glycine 2,5-furandimethyl ester before and after BOC removal.

Figure 2 shows the FTIR spectra of BOC-glycine and esters, respectively. Figure 2a shows the FTIR spectra of three BOC-glycine. In Figure 1a, the 1406 cm^−1^ absorption peak originating from -CH_3_ on the BOC group can be clearly observed due to its simple structure and chemical stability. The absorption peak at 1187 cm^−1^ originated from the stretching vibration of C–N, and the peaks at 1616 cm^−1^, 1715 cm^−1^, and 3418 cm^−1^ were originated from the bending vibration of N–H, stretching vibration of C=O, and -OH of the carboxyl group, respectively. The peak at 1650 cm^−1^ originated from the C=O of the amide bond was very small and easily overlapped with the surrounding peaks. According to Figure 1a, the results of three FTIR spectra combined with ^1^H NMR showed that BOC-glycine can be prepared under all three treatment conditions. ^1^H NMR showed that the different BOC peak areas indicated the different purity of the three BOC-glycines, with the highest purity of BOC-glycine prepared using freeze-drying. Figure 2b showed the FTIR spectra of esters before and after BOC removal. The absorption peaks of 1578 cm^−1^ and 1399 cm^−1^ were originated from the stretching vibration of C=C and the stretching vibration of C–C on the furan ring, respectively. The two peaks between 2700 cm^−1^ and 2900 cm^−1^ were attributed to the asymmetric stretching vibration of -CH_2_-. The absorption peak of 3331 cm^−1^ was attributed to N–H stretching vibration. However, the position of the absorption peak of C=O changed and the wavenumber decreased as the esterification reaction continued. Therefore, it was easily covered by other peaks in the range of 1600–1700 cm^−1^ and could not be clearly shown. In BOC-glycine 2,5-furandimethyl ester, 1400 cm^−1^ was attributed to the symmetric deformation vibration of -CH_3_ on the BOC group, but the absorption peak of 1680 cm^−1^(-NH_2_ bending vibration of primary amine) and 808 cm^−1^(-NH_2_ out-of-plane bending vibration) did not appear, as shown in 1, 2, and 3 of Figure 2b. However, after BOC was removed, the characteristic peaks of the above primary amine appeared in the glycine 2,5-furandimethyl ester prepared by using 1.875 mol/L DCC and 1.25 mol/L DCC, along with an absorption peak of about 1202 cm^−1^, which originated from the stretching vibration of C–N. Although the appearance of absorption peak of -CH_3_ was still, the intensity of the peak is significantly lower, which may be due to the fact that a small amount of BOC was not completely removed from the final product, as 4 and 5 show in Figure 2b. However, there was no significant change in the glycine 2,5-furandimethyl ester prepared by using 0.625 mol/L DCC, as shown in 3 and 6 of Figure 2b Meanwhile, BOC-glycine 2,5-furandimethyl ester prepared using 0.625 mol/L DCC could not solidify after BOC removal, and only a black viscous liquid was obtained, indicating that glycine 2,5-furandimethyl ester could not be prepared using 0.625 mol/L DCC. Combined with ^1^H NMR results, it could be seen that the spectrum of glycine 2,5-furandimethyl ester prepared using 0.625 mol/L DCC had no associated characteristic peak, which indicated that the glycine 2,5-furandimethyl ester prepared using 0.625 mol/L DCC was unsuccessful. The XRD characterization diagram of Figure 3 showed that BOC-glycine maintains a stable structure regardless of the treatment method. The structure of BOC-glycine is similar to glycine, and the position of the main peak only shifts within 1° [34]. Before and after the BOC removal experiment, the esters’ structure remained stable, and the main peak positions were almost close, mainly between 15°, 17°, and 20–22°. After BOC was removed, a new diffraction peak appeared at 18°, and the peak intensity at 23° was enhanced.

**Figure 4:**
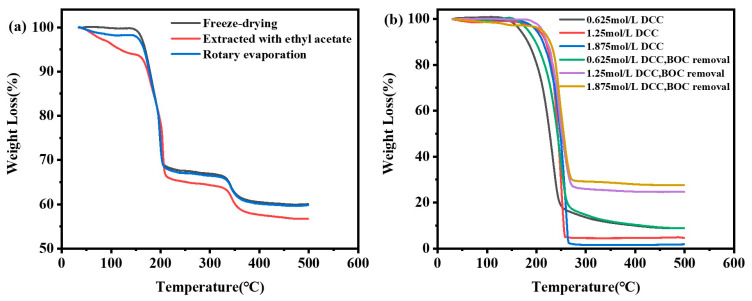
TG spectra of all prepared BOC-glycine and esters. (**a**) BOC-glycine. (**b**) Glycine 2,5-furandimethyl ester before and after BOC removal.

Figure 4 shows the TG curves of all BOC-glycine and esters. According to Figure 4a, BOC-glycine prepared using ethyl acetate extraction and vacuum drying did not show a curve platform, and the weight of BOC-glycine continuously decreased with increasing temperature, which may be related to the insufficient purity of the obtained solid. The solid prepared using the above method may contain crystal water and a small amount of unremoved ether and ethyl acetate. However, BOC-glycine prepared using rotary evaporation and freeze drying were stable before decomposition. The T_5_ (Temperature when the weight loss reaches 5%) of BOC-glycine prepared using rotary evaporation and freeze drying were 166.3 °C and 168.8 °C, respectively, which were almost the same. Although BOC-glycine prepared using ethyl acetate extraction and vacuum drying was unstable, the inflection point of the curve also appeared around 168 °C. BOC-glycine also showed a second sudden drop in the curves, which may be related to the decomposition of ammonium carbonate produced by heating the residual glycine in the solid, the temperatures shown by the three curves were all around 325 °C at this point. The TG curves of several esters were as shown in Figure 4b. It was evident that the T_5_ of BOC-glycine 2,5-furandimethyl ester and glycine 2,5-furandimethyl ester prepared by using 0.625 mol/L DCC were different from other samples. T_5_ of BOC-glycine 2,5-furandimethyl ester prepared with 0.625 mol/L DCC was 167.2 °C, which was nearly 30 °C lower than the T_5_ of BOC-glycine 2,5-furandimethyl ester prepared with 1.25 mol/L DCC (204.2 °C) and 1.875 mol/L DCC (202.1 °C). Moreover, the heat resistance of the obtained solids was improved after the BOC removal experiment. The T_5_ of glycine 2,5-furandimethyl ester prepared by using 1.25 mol/L DCC and 1.875 mol/L DCC increased by about 10 °C to 214.8 °C and 211.8 °C. However, the T_5_ of glycine 2,5-furandimethyl ester prepared using 0.625 mol/L DCC was 170.3 °C. It was obvious from the TG curves that the obtained products have good heat resistance, and all of them could withstand the temperature above 150 °C, and the ester solid was heat-resistant over 200 °C. At the same time, we tested the melting point of the obtained samples. The melting points of the three BOC-glycines were all around 100–110 °C, and the melting points of glycine 2,5-furandimethyl ester prepared by using 1.25 mol/L DCC and 1.875 mol/L DCC were all between 168 °C and 179 °C.

**Figure 5:**
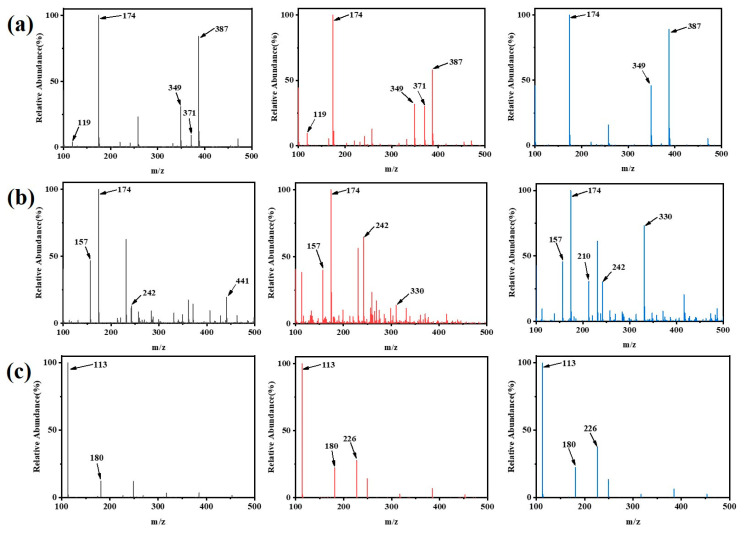
LC–MS spectra of all prepared BOC-glycine and esters. (**a**) BOC-glycine. Black line: Freeze-drying. Red line: Vacuum distillation. Blue line: Extracted with ethyl acetate after filtration and dried in vacuum. (**b**,**c**) Glycine 2,5-furandimethyl ester before and after BOC removal. Black line: Ester used 0.625 mol/L DCC. Red line: Ester used 1.25 mol/L DCC. Blue line: Ester used 1.875 mol/L DCC.

Figure 5 shows the LC–MS spectrum of several BOC-glycine and esters. It was obvious that the mass spectrum Figure 5a of BOC-glycine that the peak at 174 *m/z* belonged to [M − H]^−^, while the peaks of [2M + K]^+^, [2M + Na]^+^, and [2M + H]^+^ were located at 387 *m/z*, 371 *m/z*, and 349 *m/z*, and there were few other peaks in the spectrum. According to the MS spectrum of BOC-glycine 2,5-furandimethyl ester, it was evident that BOC-glycine 2,5-furandimethyl ester prepared by using 0.625 mol/L DCC appears [M − H]^−^ at 441 *m/z*, but there were not many fragment ions related to BOC-glycine 2,5-furandimethyl ester, and the abundance was not high, only [M-BOC]^+^. In addition to the two peaks above, the other two BOC-glycine 2,5-furandimethyl esters also had [M − C(CH_3_)_3_ ]^+^ (330 *m/z*) and [M − BOC − NH_2_]^+^ (210 *m/z*), and the abundance of the peaks was higher than that of BOC-glycine 2,5-furandimethyl ester prepared using 0.625 mol/L DCC. In addition, the peak of 157 *m/z* may be attributed to the dehydroxylation of BOC-glycine or the fragment ion of DMSO solvent, as shown in Figure 5b. After BOC removal experiment, the MS spectrum also showed the corresponding fragment ions. Among them, 113 *m/z* belonged to glycine fragment ion [M + K]^+^ in glycine 2,5-furandimethyl ester, while [M − CH_2_NH_2_]^+^ in glycine 2,5-furandimethyl ester belonged to 180 *m/z*. The peak of [M − NH_2_]^+^ appeared in glycine 2,5-furandimethyl ester prepared by using 1.25 mol/L DCC and 1.875 mol/L DCC, and its position was 226 *m/z*. According to Figure 5c, the latter two kinds of glycine 2,5-furandimethyl esters had more fragment ions and higher abundance than the first one.

### 3.2. Attempt of Application of Glycine 2,5-Furandimethyl Ester

**Figure 6:**
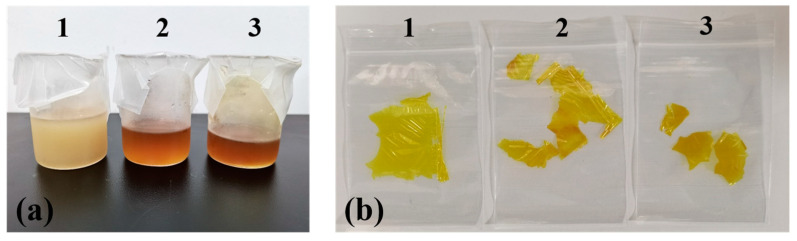
The prepared polyimide colloid (**a**) and membranes (**b**). **1.** 5%, 20 wt%; **2.** 10%, 20 wt%; **3.** 10%, 25 wt%.

To explore the application of glycine 2,5-furandimethyl ester in polyimide synthesis, we tried to synthesize three polyimides by adding glycine 2,5-furandimethyl ester prepared using 1.25 mol/L DCC as diamine. The detailed composition is shown in Table 1. Due to the addition of glycine 2,5-furandimethyl ester, the prepared polyimide colloid was yellow, and the color became darker with the addition of glycine 2,5-furandimethyl ester, as shown in Figure 6a. Furthermore, we prepared membranes from polyimide colloid and characterized them using TG and SEM. The appearance of three membranes were all yellow and transparent with good light transmission. However, the prepared membrane was brittle, the toughness was lower than the polyimide membrane that was prepared only using PMDA and ODA. This was because the furan ring’s rigidity of glycine 2,5-furandimethyl ester was not as good as benzene ring, which led to a decrease in mechanical properties. After the addition of glycine 2,5-furandimethyl ester was reduced, the mechanical properties and the integrity of the membrane after imidization was improved. The appearance of the prepared membranes was shown in Figure 6b. At the same time, the degree of polymerization of the prepared polyimide was also good. As presented in Table 1, the maximum weight average molecular weight of the three polyimides all exceeded 350,000, with a maximum close to 560,000. In addition, the increase dosage of diamine and dianhydride such as glycine 2,5-furandimethyl ester contributed to the increase in average molecular weight to some extent.

**Figure 7:**
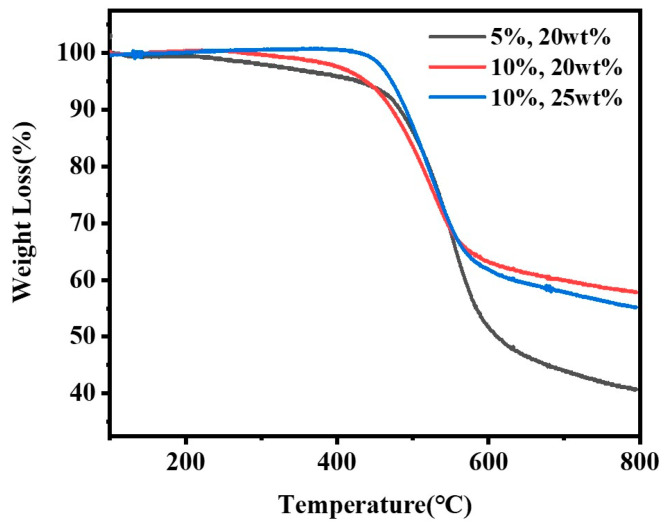
TG spectra of all prepared polyimide membranes.

The thermogravimetric characteristics of three polyimide membranes are shown in Figure 7. As can be seen from the curves, the T_5_ of 5%, 20 wt% and 10%, 20 wt% were similar, reaching 459.3 °C and 477.8 °C, respectively, while the T_5_ of 10%, 25 wt% was greatly improved, reaching 502 °C. At 800 °C, the residual weight of three polyimide membranes were all above 40%, and those of 10%, 20 wt% and 10%, 25 wt% were above 50%. Compared with the T_5_ (571.3 °C) of the polyimide membrane prepared only using PMDA and ODA [35], the T_5_ value of the polyimide membrane prepared with glycine 2,5-furandimethyl ester decreased slightly, which due to the fact that the thermal stability of the furan ring was less stable than the benzene ring. Moreover, the presence of impurities possibly reduced the purity of the obtained glycine 2,5-furandimethyl ester. In general, the polyimide membrane prepared with glycine 2,5-furandimethyl ester had good heat resistance, and the temperature resistance may be close to that of pure polyimide by increasing the purity and adding fillers.

**Figure 8:**
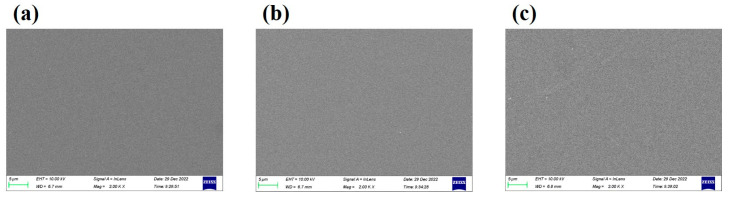
SEM images of the surface of all prepared polyimide membranes. (**a**) 5%, 20 wt%; (**b**) 10%, 20 wt%; (**c**) 5%, 20 wt%.

The surface morphology of polyimide membrane was characterized using SEM. Compared with the surface morphology of the polyimide membrane prepared only using PMDA and ODA [35], the polyimide membrane prepared with glycine 2,5-furandimethyl ester and ODA as diamines also were also essentially free of pores and particles, and had a relatively uniform surface. A small amount of impurities in glycine 2,5-furandimethyl ester had no significant effect on the membrane formation of polyimide. This showed that polyimide could also be prepared with glycine 2,5-furandimethyl ester as partial diamine. The surface morphology of three kinds of polyimide membranes was shown in Figure 8.

## 4. Conclusions

In this work, three BOC-glycines, BOC- glycine 2,5-furan dimethyl esters, and glycine 2,5-furan dimethyl esters were prepared by changing different experimental contents, and they were characterized using FTIR and TG, etc. The results showed that BOC-glycine could be prepared using three different posttreatment methods, differing only in purity. However, the final diamine could not be prepared by using 0.625 mol/L DCC, which was due to the insufficient amount of DCC for the esterification reaction to proceed efficiently. The final target product can be prepared by using 1.25 mol/L DCC and 1.875 mol/L DCC. In addition, we also used the prepared glycine 2,5-furan dimethyl ester to synthesize polyimide. Since the furan ring was not as rigid as the benzene ring, glycine 2,5-furan dimethyl ester cannot completely replace ODA for the preparation of polyimide temporarily. From the characterization results, the obtained polyimide had moderate molecular weight and good thermal stability, and its surface morphology was consistent with that of polyimide prepared only by PMDA and ODA. Although it was only a simple attempt to apply furan diamine to the synthesis of polyimide at present, with the deepening of the concept of environmental protection and energy saving, furan diamine will play a more important role in the synthesis of polyimide in the future.

## Figures and Tables

**Table 1:** Three groups of prepared polyimide.

Number	The Dosage of Diamine/g		The Dosage of Dianhydride/g	The Dosage of Solvent/g	Maximum Molecular Weight Average/Mw	Name
	Glycine 2,5-Furandimethyl Ester	ODA	PMDA	DMAc		
1	0.1924(5 wt%)	3.6552(95 wt%)	4.1524	32(80 wt%)	392,046	5%, 20 wt%
2	0.3867(10 wt%)	3.4806(90 wt%)	4.1327	32(80 wt%)	459,361	10%, 20 wt%
3	0.4809(10 wt%)	4.3286(90 wt%)	5.1905	30(75 wt%)	563,731	10%, 25 wt%

## Data Availability

All the research data have been completely presented in the article.
